# Domestic animals infected with *Mycobacterium ulcerans*—Implications for transmission to humans

**DOI:** 10.1371/journal.pntd.0006572

**Published:** 2018-07-02

**Authors:** Rousseau Djouaka, Francis Zeukeng, Jude Daiga Bigoga, Solange E. Kakou-Ngazoa, Romaric Akoton, Genevieve Tchigossou, David N’golo Coulibaly, Sodjinin Jean-Eudes Tchebe, Sylla Aboubacar, Clavella Nantcho Nguepdjo, Eric Tossou, Razack Adeoti, Thèrèse Marie Ngo Nsonga, Yao Akpo, Innocent Djegbe, Manuele Tamo, Wilfred Fon Mbacham, Anthony Ablordey

**Affiliations:** 1 The AgroEcoHealth Platform, International Institute of Tropical Agriculture (IITA), Cotonou, Bénin; 2 Faculty of Science, Department of Biochemistry, University of Yaoundé I, Yaoundé, Cameroon; 3 Department of Technics and Technology, Platform of Molecular Biology, Pasteur Institute Abidjan, Abidjan, Côte d’Ivoire; 4 University of Abomey-Calavi, Faculty of Science and Technics, Calavi, Benin; 5 Akonolinga District Hospital, Akonolinga, Cameroon; 6 Faculty of Agronomy, University of Parakou, Laboratory of Ecology, Health and Animal Production (LESPA), Parakou, Bénin; 7 Department of Bacteriology, Noguchi Memorial Institute for Medical Research, University of Ghana, Legon, Accra, Ghana; University of California San Diego School of Medicine, UNITED STATES

## Abstract

**Background:**

The environmental pathogen, *Mycobacterium ulcerans* (MU) can infect both humans and animals and cause Buruli ulcer (BU) disease. However, its mode(s) of transmission from the colonized environment to human/animal hosts remain unclear. In Australia, MU can infect both wildlife and domestic mammals. Till date, BU-like lesions have only been reported in wildlife in Africa. This warrants a thorough assessment of possible MU in domestic animals in Africa. Here, we screened roaming domesticated animals that share the human microhabitat in two different BU endemic sites, Sedje-Denou in Benin and Akonolinga in Cameroon, for MU lesions.

**Methodology/Principal findings:**

We screened roaming mammals and birds across 3 endemic villages of Sedje-Denou in Southern Benin and 6 endemic villages of Akonolinga in Cameroon. After approval from relevant authorities, specimens (wound swabs and tissue fragments) were collected from animals with open or active lesion and systematically screened to detect the presence of MU though the diagnostic DNA targets IS*2404*, IS*2606* and KR-B. Out of 397 animals surveyed in Akonolinga, 44 (11.08%) carried skin lesions and all were negative for MU DNA. For Sedje-Denou, only 25 (6.93%) out of 361 animals surveyed carried external skin lesions of which 2 (8%) were positive for MU DNA targets. These MU infected lesions were found in two different villages on a goat (abdominal part) and on a dog (nape area of the neck). Source-tracking of MU isolates within infected animal lesions was performed using VNTR genotyping and further confirmed with sequencing. One MU VNTR genotype (Z) was successfully typed from the goat lesion. The evolutionary history inferred from sequenced data revealed a clustering of animal MU isolates within isolates from human lesions.

**Conclusion/Significance:**

This study describes the first report of two MU infected lesions in domestic animals in Africa. Their DNA sequence analyses show close relationship to isolates from human cases. It suggests that MU infection should be suspected in domestic hosts and these could play a role in transmission. The findings further support the hypothesis that MU is a ubiquitous environmental pathogen found in endemic areas, and probably involved in a multiple transmission pathway.

## Author summary

Buruli ulcer (BU) remains a major Public Health problem in rural communities in sub-Saharan Africa. There are several reports of the occurrence of BU in Wildlife as well as domestic animals in Australia leading to the suggestion that animals may play a role in the transmission of MU to humans. Report of BU in animals is however scanty in Africa and no significant link has been made between BU in humans and animals. BU-like lesions were investigated in 397 and 361 roaming domestic animals respectively from Sedje-Denou and Akonolinga. Wound swabs, and tissue fragments were collected from animals with active lesions. Overall, 2 (8%) type I (<5 cm) animal lesions (localized on the abdominal part of a goat and the nape area of a dog) were colonized by MU in Benin. MU VNTR genotypes Z (4, 1, 2, 2) and C- (3, 1, 2, 0) were identified in the lesions of the goat and dog respectively. Significant homology was found between orthologous sequences of MU strains infecting animals and humans. The evolutionary history inferred from sequenced data revealed a clustering of animal MU isolates within isolates from human lesions. New reservoirs of MU were found through this study and allowed to a new interpretation of the life cycle of this mycobacterium from the risk environment to humans in Africa.

## Introduction

Buruli ulcer (BU), the necrotizing skin disease caused by *M*. *ulcerans* (MU) remains a major Public Health problem in more than 33 countries worldwide. Although the distribution of BU is global, its burden is highest in remote communities of West and Central African regions including Benin, Ghana, Ivory Coast, Democratic Republic of Congo and Cameroon; as well as some coastal localities of Australia. These African endemic areas recently reported the highest incidence of the disease with 89% of new cases reported in 2014 [1,2]. They also share the most pathogenic MU isolates which are characterized by the production of the polyketide-derived macrolide toxin mycolactone A/B, the main virulence factor for MU [3].

Mycobacteria are the etiology of important diseases in humans and a wide range of animals including cattle, sheep, goats, deer, possums, badger, elephants, dogs, cats, birds, amphibians, and fishes. [4]. *Mycobacterium sp*. such as *M*. *bovis* are pathogens that have become important agents at the interface of humans, domestic livestock and wildlife [5]. Animals such as rodents (rats, mice and shrews), monkeys, guinea pigs, rabbits and armadillos are sensitive to experimental infection with MU and constitute good models for the study of BU disease [6,7]. However, humans were considered the only mammalian host of the disease. Animal cases of BU were reported in Australia in both wildlife (possums, alpacas and koalas) and domestic mammals (cats, dogs and horses) [8–12]. The possible role of animals in BU transmission has also been suggested by the positive correlation observed between the prevalence of MU DNA in possum feces and BU endemicity in Australia [9]. Thus, domestic animals could play a significant role as amplifiers of MU pathogen, influencing the transmission cycle of human infectious disease [10,13]. Hence, potential colonization of the domestic microenvironment by MU emphasizes the risks associated with the close contact between humans and peridomestic animals.

A tentative mode of transmission involving both humans and animals has been postulated [10]. This model suggests that domestic animals can be infected when feeding in the infected environment, or, by simple contact with the soils or feces of wild animals. Humans can later be infected through direct contact with animal excreta, animal bites or through ectoparasites [10,14,15]. In some instances, BU infected patients with poor hygiene conditions may play an active role in the dissemination and distribution of the *Mycobacterium* in the environment [16], and indirectly favor the focal distribution of the disease in new hosts such as animal hosts.

On the African continent on the other hand, there are relatively few reports infection in animals. MU DNA has been detected only in small mammals such as mice (*Mastomys sp*.) with ulcerative lesions [17,18]. A study carried out in Benin by Durnez et al. [14] revealed a wide distribution of *Mycobacterium sp*. in terrestrial small animals (mice, shrews and rats) and insectivores, but not of MU. Although MU was detected in the feces of a peridomestic small mammal, *Thrynomys swinderianus* (agouti) in Ivory Coast [19], it was completely absent in a wide range of fecal materials from domestic animals and humans in endemic villages of Ghana [20,21]. More significantly, domestic animals have not been investigated for BU infection. Thus, it is not yet known whether in Africa domestic animals play a role in the environmental cycle of MU as suggested for wildlife and small mammals in Australia. [17,18].

Humans and mammals (as well as amphibians—turtles) not only carry the bacteria but also develop active disease. Open sores and ulcers could represent a potential source of contamination and transmission to other hosts [8,9,11,12,16–18,22]. However, it is still unclear whether BU infected animals can develop the spectrum of human disease.

Thus, we hypothesize in this studies that the overlapping ecology of human and animal habitats could favor the transmission of MU. We employed molecular tools to screen BU like-lesions in roaming domestic animals (carnivorous, cattle and domesticated birds) sharing the same habitat with humans in endemic localities of Sedje-Denou in Benin (West Africa) and Akonolinga in Cameroon (Central Africa). In these regions, domestic livestock constitutes one of the main resources of the communities, which mainly survive by subsistence agriculture, fishing, breeding, hunting and informal commerce.

## Materials and methods

### Ethical guidelines

Approval for animal trapping and samples collection were obtained from the International Institute of Tropical Agriculture (IITA-Benin) and the Cameroon National Ethics Review Committee (N°946/CE/CNERSH/SP). Additional permits were also sought from the local leaders of Sedje-Denou and Akonolinga districts in Benin and Cameroon respectively. No invasive procedure was performed on animals, and all animals carrying active external lesions were systematically treated free of charge by a veterinarian physician. Community consent was also obtained prior to animal’s surveys and owners helped to trap animals in each investigated community. All activities on animals were conducted in compliance with the national animal protection law (MEPN-Benin and MINFOF-Cameroon) in addition to other international guidelines.

### Study site

**Fig 1:**
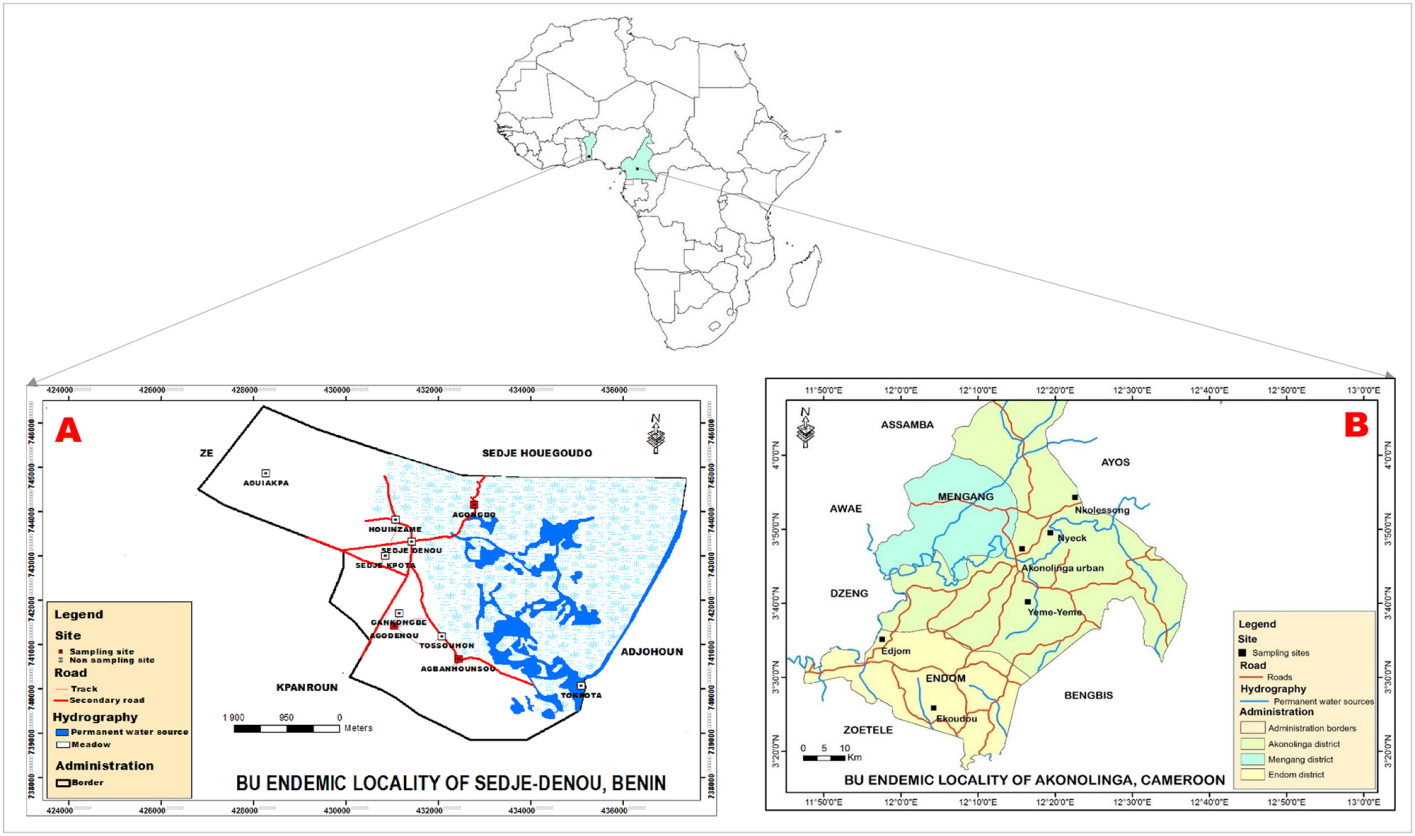
Location of the sampling sites in Western and Central Africa. **A)** BU endemic locality of Sedje-Denou in Benin; **B)** BU endemic locality of Akonolinga in Cameroon.

This study was conducted in two BU endemic areas in West and Central Africa (Fig 1). The endemic area of Sedje-Denou (6° 32’ N and 2° 13’ E) is located in the Southern part of Benin in West Africa. Sedje-Denou is a locality under the Commune of Ze, which is the second most BU endemic locality in Benin with a prevalence of 0.45% [23]. The presence of rivers and wetlands make the locality a typical environment for MU. The region is bordered to the East by the Oueme River which has several tributaries across the villages with the creation of meadows and wet march lands. Risk factors for BU in the region include non-protection from swamps, BCG-vaccinated patients (>5 years old), living or participating in agricultural activities near the river and improper care for wounds [24,25]. The predominant occupation of the inhabitants is farming and fishing. Three endemic villages namely Agbahounsou, Agodenou and Agongbo with a history of high burden of disease were investigated in this region. The specific study sites were chosen because of endemicity of BU and ecological risk factors, proximity to the Oueme River (main BU risk factor) and the meadow and march land which constitute water supply sources for roaming domestic animals from the villages [26].

The BU endemic area of Akonolinga (3° 46’ N and 12° 15’ E) is in the Central region of Cameroon in Central Africa. Akonolinga health district is at approximately 70 km East to the West, and a 100 km North to South, with a surface of approximately 4500 km^2^ [27]. Subsistent farming, fishing and hunting are the main occupation of inhabitants of Akononlinga. Akonolinga is the most endemic area for BU in Cameroon. The disease prevalence in the region is high and ranges between 0.41–0.73% (average 105 cases) per year [27]. The district located in the Nyong valley is crossed by the Nyong River which has several tributaries throughout the villages. Risk factors for BU in the region include wading in swamps, living near cocoa plantations or wood, having activities in the Nyong basin, and improper care for wounds [28,29]. Six high endemic villages, Akonolinga urban, Edjom, Ekoudou, Nyeck, Nkolessong, and Yeme-Yeme were selected as study sites based on published epidemiological data and ecological characteristics favoring possible MU distribution, and proximity with the Nyong River, which constitutes the main water supply source for domestic animals from these communities.

### Animal trapping

Field activities were stratified into two steps: a 2 days sensitization activity in the communities during which a community aid was trained in each village; and a survey step which lasted for about 2 to 3 days in each village. Prior to the community surveys roaming animals living in close habitat with humans were kept in a paddock. Animals were kept enclosed till the arrival of the research team in the houses where they were systematically screened for lesions.

### Animal identification and samples collection

The trapped animals were identified as cattle (goat, sheep and cow), carnivores (dog and cat), omnivores (pig) and domesticated birds (chicken and duck). Identified animals were further examined physically by palpation for the presence of any skin lesion or nodule. Sex and age were recorded, and the animals were marked to avoid recaptures. Wound swab and tissue fragments were systematically collected in duplicate from animals carrying skin/soft tissue lesion or open sore as previously described [18,19]. Characteristic BU lesions as well as those not likely to be BU lesions were investigated. After sample collection the lesions were cleaned, disinfected and treated by a trained veterinarian doctor. After clinical examination, a differential diagnosis was done by the veterinarian and antibiotic spray (VETOSPRAY Lot H-011, France) and dermal antiseptic (Betadine Lot 31650, MEDIA Manufacturing, Paris, France) were applied accordingly to stop the propagation of colonized microbial pathogens. For lesions suspicious of BU, the veterinarian applied a combination of rifampicin 10 mg/kg and streptomycin 15 mg/kg. In addition, animals were systematically treated with 10 mg/ml ivermectin injection 1% (Lot 1308211–01, Alfamec, WOERDEN-Holland) to protect against any parasitic infection.

### Sample processing and extraction of genomic DNA

Overall, 250 μl of each homogenate (swabs and tissue fragments) was used for DNA extraction. Total genomic DNA (gDNA) was extracted using the Qiagen DNasy Blood and Tissue Kit according to the manufacturer`s instructions (Qiagen). Negative controls (nuclease-free water, Sigma-Aldrich) were added at a frequency of 10% (1 control per batch of 10 extractions) to monitor potential cross-contaminations.

### Real time PCR amplifications

TaqMan real time quantitative PCR analysis was performed on DNA extracts to screen for the presence of MU DNA according to Fyfe et al. [30]. Although no internal positive control (IPC) was included in the analyses, IS*2404* negative samples were diluted 1/10 and retested for the detection of PCR inhibitors. All samples positive for the IS*2606* and KR-B targets were tested in duplicate for data validation. Two positive controls (*MU* Agy99 DNA) as well as two no-template negative controls (nuclease-free water, Sigma-Aldrich) were used to guide the experiments against false positive and negative results.

### MIRU-VNTR profiling

Variable Number Tandem Repeat (VNTR) typing of four VNTR loci (MIRU1, Locus 6, VNTR19 and ST1) was performed as a confirmatory assay for all MU positive samples to decipher the MU strains and differentiate them from other mycolactone producing mycobacteria (MPMs). VNTR genotyping was performed according to Williamson et al. [31]. Briefly, 5 μl of gDNA extract were amplified in 45μl PCR mixture consisting of 1X Flexi Taq-Polymerase buffer (Promega, Germany), 0.4μM of each specific primer, 0.2 mM dNTP-mix, 1.5 mM MgCl_2_, 1U Go-Flexi Taq-polymerase (Promega, Germany), and sterile DNase-free water (Sigma-Aldrich). The amplification process was performed in GenAmp PCR System 9700 (Applied Biosystems). After an initial denaturation at 95°C for 2 min; MIRU1, locus 6 and VNTR 19 loci were amplified by 40 cycles of 1 min steps respectively at 94°C, 58°C, and 72°C; and one last elongation step of 10 min at 72°C. The ST1 locus was amplified using the same conditions with 30 s denaturation at 94°C and 30 s primer-hybridization at 65°C as described by Hilty et al. [32]. The amplicons were separated on 1.5% agarose gel stained with Midorin Green Advance DNA Stain (Nippon Genetics, Europ GmbH), and visualized under a UV light source (UVP, Benchtop Variable Trans-illuminator, Cambridge, UK). Amplicons band sizes were analyzed by comparing to a 100bp DNA molecular ladder (Promega, Germany) and the VNTR repeat/copy numbers were estimated as previously described [32–34]. VNTR allelic profiles were defined according to the copy number of each amplified locus and were in the order of MIRU1, Locus 6, ST1 and VNTR 19. BU patient specimens (MU positive swabs) from Benin and Cameroon were also genotyped to source-tack MU strains from the environment to humans. Genomic DNA of MU Agy99 was subsequently tested in duplicates as positive control. All PCR runs also included negative controls (sterile DNase-free water, Sigma-Aldrich) for quality control and for detection of potential contaminations. Negative samples were treated as above.

### Genomic sequence analysis

Representative amplicons of amplified VNTR loci were confirmed with sequencing using the forward and reverse primers at the Molecular Biology Platform of Pasteur Institute (Ivory Coast). Products of the expected size were purified using the QIAquick Gel Extraction Kit according to the manufacturer`s instructions (Qiagen). Purified PCR products were subjected to BigDye Terminator v3.1 Cycle Sequencing Kit (for Sanger sequencing) in GenAmp PCR System 9700 (Applied Biosystems) according to the manufacturer’s instructions (Applied Biosystems). Sequence products were thereafter subjected to purification using the Dye Terminator Removal Kit according to the manufacturer`s instructions (Thermo Fisher Scientific). This second purification step was designed to remove unbound fluorescence labeled dideoxyribonucleotides (ddNTPs) and excess salt from sequence reactions prior to sequence analysis. Cleaned sequences were analyzed in ABI 3500xL Genetic Analyzer using the ABI9500 sequencing program (Applied Biosystems).

### Data analysis

Sequence data were edited in MEGA 6.0 software and consensus sequences generated in FASTA format [35]. Multiple sequence alignment (MSA) of consensus data was performed in NCBI-BLAST and the evolutionary history (phylogenetic analysis) of MU isolates was inferred using the UPGMA method within MEGA 6.0 [36]. Reference sequences of MIRU1 orthologs were retrieved from GenBank.

## Results

### Distribution of roaming domestic animals within communities

A total of 361 domestic animals roaming around the human habitats were systematically investigated in the 3 endemic villages in Benin, with 161 (44.6%) in Agodenou, 109 (31.19%) in Agongbo and 91 (25.21%) in Agbahounsou. Animal diversity includes carnivore species, bovidae species and bird’s species. Most animals investigated were among the Bovidae (cattle) family including sheep (*Ovis aries*, 29.09%), goat (*Capra aegagrus hircus*, 18.56%) and cow (*Bos Taurus*, 0.55%). Different species of carnivores were identified including dog (*Canis lupus familiaris*, 4.43%) and cat (*Felis catus*, 3.05%). Pig (*Sus scrofa domesticus*, 6.93%) constituted the only omnivore identified. Two bird species were identified in the localities namely chicken (*Gallus gallus domesticus*, 29.36%) and duck (*Anas platyrhynchos sp*., 8.03%). Skin lesions (open sores) were observed in 25 (6.93%) roaming domestic animals in the 3 endemic villages in Benin. Identified lesions were on the head, ear, abdomen, foot (tight), tail, and the nape area of the neck; and 9 (36%) of these were BU-like (S1 Fig). There was no specificity on lesions distribution according to animal and animal body parts. However, most of the lesions (98.8%) were observed on the head, abdomen and the foot of animals. Dogs (4/16, 25%), goats (10/67, 14.93%), ducks (2/29, 6.9%), sheep (7/105, 6.67%), pigs (1/25, 4%) and chickens (1/106, 0.94%) carried wound in the areas studied in Benin.

Overall, 397 roaming animals were systematically investigated with 118 (29.72%) in Edjom, 75 (18.89%) in Nyeck, 66 (16.63%) in Yeme-Yeme, 55 (13.85%) in Ekoudou, 50 (12.59%) in Akonolinga urban center, and 33 (8.31%) in Nkolessong. Distribution of animals in the two study sites were similar. Forty-four (11.08%) roaming domestic animals investigated in the 6 endemic villages in Cameroon carried at least one active external lesion. Identified lesions here were on the head, ear, tail, abdomen, leg, nape area of the neck, lateral side, back, and the sex of the animal; and 7 (15.91%) of these were BU-like (S1 Fig). As observed in Benin, up to 96% of the lesions appeared on the head, abdomen and the foot. Lesions distribution varied among animals and the wounded animals include dogs (30/54, 55.56%), goats (8/73, 10.96%), ducks (2/40, 5%), sheep (2/47, 4.26%) and pigs (2/66, 3.03%).

### Detection of mycobacteria/*M*. *ulcerans* in animals presenting external lesions within communities

Quantitative PCR (qPCR) analysis of IS*2404* molecular markers performed on animal specimens detected bacteria DNA in the lesions of 9 (36%) animals out of 25 found in endemic villages in Benin. Five (55.56%) of the IS*2404* positive specimens also tested positive for IS*2606*. whereas only 2 (22.22%) of the IS*2404*-positive lesions were positive to the ketoreductase B domain of MU plasmid pMUM001. Overall, 2 (8%) external lesions tested positive for all three targets, IS*2404*, IS*2606* and KR-B suggesting the presence of MU. MU infected lesions were therefore identified in two animals including one goat and one dog in Benin. Details on the distribution of MU targets according to animal species and BU locality in Benin are shown in Table 1. ΔCts (IS*2606*-IS*2404*) values of 2.62 and 2.83 cycles were obtained from the dog and goat lesion respectively, confirming the presence of MU in these lesions as previously defined by Fyfe et al., 2007 [30].

**Table 1:** Distribution of MU DNA targets in animal lesions according to animal species and BU locality in Benin.

Country	Locality	Animal carrying external lesions	qPCR diagnostics for *M*. *ulcerans*			*M*. *ulcerans distribution*
			*N*° *IS2404 positive/total sampled (%)*	*N*° *IS2606 positive/total sampled (%)*	*N*° *KR-B positive/total sampled (%)*	
Benin	Agongbo	**Dog**	**2/3 (66.67%)**	**1/2 (50%)**	**1/2 (50%)**	**1/3 (33.33%)**
		Goat	2/5 (40%)	2/2 (100%)	0/2 (0%)	0/5 (0%)
		Sheep	1/4 (25%)	0/1 (0%)	0/1 (0%)	0/4 (0%)
		Duck	0/1 (0%)	0/0 (0%)	0/0 (0%)	0/1 (0%)
	Agodenou	Dog	0/1 (0%)	0/0 (0%)	0/0 (0%)	0/1 (0%)
		Goat	1/3 (33.33%)	0/1 (0%)	0/1 (0%)	0/3 (0%)
	Agbahounsou	**Goat**	**1/2 (50%)**	**1/1 (100%)**	**1/1 (100%)**	**1/2 (50%)**
		Sheep	1/3 (33.33%)	1/1 (100%)	0/1 (0%)	0/3 (0%)
		Pig	0/1 (0%)	0/0 (0%)	0/0 (0%)	0/1 (0%)
		Chicken	0/1 (0%)	0/0 (0%)	0/0 (0%)	0/1 (0%)
		Duck	1/1 (100%)	0/1 (0%)	0/1 (0%)	0/1 (0%)
**Total**			**9/25 (36%)**	**5/9 (55.56%)**	**2/9 (22.22%)**	**2/25 (8%)**

In Cameroon, none of the 44 external lesion specimens subjected to qPCR analysis revealed the presence of MU. Eleven (25%) lesions tested positive for IS*2404*, whereas the IS*2606* marker was detected in only 3 (27.27%) IS*2404*-positive lesions. None of these specimens was positive to the KR-B domain in the 6 endemic villages investigated in Akonolinga. Details on the distribution of MU targets according to animal species and BU locality in Cameroon are showed in Table 2.

**Table 2:** Distribution of MU DNA targets in animal lesions according to animal species and BU locality in Cameroon.

Country	Locality	Animal carrying external lesions	qPCR diagnostics for M. ulcerans			*M*. *ulcerans distribution*
			*N*° *IS2404 positive/total sampled (%)*	*N*° *IS2606 positive/total sampled (%)*	*N*° *KR-B positive/total sampled (%)*	
Cameroon	Yeme-Yeme	Dog	1/2 (50%)	0/1 (0%)	0/1 (0%)	0/2 (0%)
		Goat	0/2 (0%)	0/0 (0%)	0/0 (0%)	0/2 (0%)
		Duck	0/1 (0%)	0/0 (0%)	0/0 (0%)	0/1 (0%)
	Edjom	Dog	2/6 (33.33%)	1/2 (50%)	0/2 (0%)	0/6 (0%)
		Sheep	0/1 (0%)	0/0 (0%)	0/0 (0%)	0/1 (0%)
	Akonolinga urban	Dog	4/17 (23.53)	1/4 (25%)	0/4 (0%)	0/17 (0%)
		Goat	1/4 (25%)	0/1 (0%)	0/1 (0%)	0/4 (0%)
		Sheep	0/1 (0%)	0/0 (0%)	0/0 (0%)	0/1 (0%)
	Ekoudou	Dog	1/2 (50%)	0/1 (0%)	0/1 (0%)	0/2 (0%)
		Goat	0/1 (0%)	0/0 (0%)	0/0 (0%)	0/1 (0%)
		Pig	1/2 (50%)	1/1 (100%)	0/1 (0%)	0/2 (0%)
	Nkolessong	Dog	0/2 (0%)	0/0 (0%)	0/0 (0%)	0/2 (0%)
	Nyeck	Dog	0/1 (0%)	0/0 (0%)	0/0 (0%)	0/1 (0%)
		Goat	0/1 (0%)	0/0 (0%)	0/0 (0%)	0/1 (0%)
		Duck	1/1 (100%)	0/1 (0%)	0/1 (0%)	0/1 (0%)
**Total**			**11/44 (25%)**	**3/11 (27.27%)**	**0/11 (0%)**	**0/44 (0%)**

### Clinical description of *M*. *ulcerans* infected lesions in animals within communities

#### MU infected lesion found in one goat at Agbahounsou

**Fig 2:**
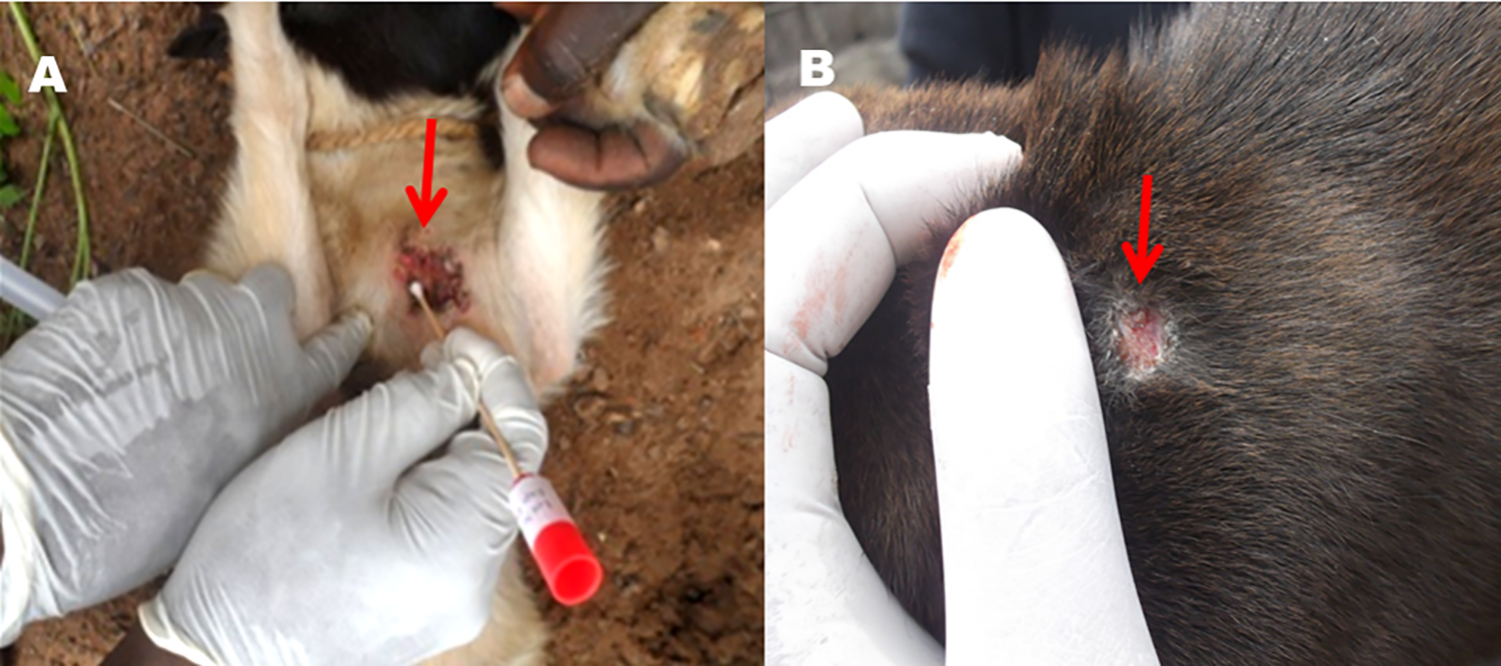
BU-like infected lesions in roaming domesticated animals. The lesions are shown as captured before any treatment on infected animals. **A) MU infected lesion on the abdominal part of a 3 years old female goat**. This BU-like lesion (1.8 cm diameter) appears reddish with undermined borders, a well circumscribed ulceration and a necrotic base. **B) MU infected lesion on the nape area of the neck of a 20 months aged female dog**. The type 1 lesion (1.4 cm diameter) characteristic of BU appears reddish in the center and whitish at the borders. The borders of this well-circumscribed lesion remain undermined.

In the BU endemic village of Agbahounsou in Southern Benin, a female goat aged 3-year-old carried a MU positive lesion on the abdomen (Fig 2A). The BU like lesion (1.8 cm diameter, type 1 lesion) appeared reddish with undermined borders, a well-circumscribed ulceration and a necrotic base. The overall animal body conditions were fair and one additional non-infected lesion was found on the right hind leg (tight). MU-Positive specimens collected from this lesion include both the wound swabs and tissue fragments as detailed in Table 3.

**Table 3:** Molecular diagnosis and genotyping of MU isolates from animal and patient lesions within BU endemic regions.

Country	Locality	Animal common name	Nature of sample	Sample code	qPCR diagnostics for *M*. *ulcerans*			MIRU-VNTR genotyping (copies detected)				MIRU-VNTR genotype
					IS*2404* (Ct)	IS*2606* (Ct)	KR B (Ct)	MIRU 1	Locus 6	ST1	VNTR 19	
Ghana (Control)	NMIMR	Human	Agy99 isolate	MUAgy99	20.97	26.07	25.24	3	1	2	2	Cˆ
Benin	Sedje Health Center	Human	WS	MHC15S	28.65	33.01	32.13	3	1	2	2	Cˆ
Benin	Sedje Health Center	Human	WS	MHC16S	29.94	34.2	35.25	3	1	2	2	Cˆ
Cameroon	Akonolinga-Hospital	Human	WS	MHC17A	27.5	31.19	32.04	3	1	2	2	Cˆ
Cameroon	Akonolinga-Hospital	Human	WS	MHC18A	28.97	32	31.9	3	1	2	2	Cˆ
Benin	Agongbo	Dog	TF	AD01L	30.3	32.92	38.24	3	1	2	0	C-*
Benin	Agbahounsou	Goat	WS	AD80Sw	29.07	31.9	36.23	4	1	2	2	Z~
			TF	AD81L	30.17	33.1	35.39	4	1	2	2	Z~

Letters carrying the same symbols (^ or ~) are similar genotypes.

* Incomplete genotype with lack of VNTR19. WS: Wound swabs, TF: Tissue fragments.

#### MU infected lesion found in one dog at Agongbo

The second MU infected lesion was found in a 24 months aged female dog in the BU-endemic village of Agongbo in Southern Benin. This lesion characteristic of BU (1.4 cm diameter) appeared on the nape area of the animal’s neck (Fig 2B). The type 1 lesion appeared reddish at the center and whitish at the borders. The borders of this well-circumscribed lesion remained undermined. The animal was otherwise healthy and there were no additional lesions on the rest of the body. From the two types of specimens collected, only the tissue fragment samples tested positive for MU. (Table 3).

### MIRU-VNTR genotyping of MU isolates in animal samples

MIRU-VNTR analysis was performed for MU-positive and MU-negative animal specimens to confirm the BU diagnosis and infer the evolution history between isolates. Allelic profiles/genotypes were written in the sequential order of (MIRU1, Locus 6, ST1, and VNTR 19) according to the copy numbers of each amplified locus. Overall, genotypes for MU-negative specimens were undetermined because of the heterogeneity in loci distribution and the non-amplification of some VNTR loci (S1 Table). The heterogeneity among VNTR loci further confirms the absence of MU in these negative samples. Concerning the genotyping process of positive lesions, the percentage distribution of VNTR loci were as MIRU1 (3/3, 100%), Locus 6 (3/3, 100%), ST1 (3/3, 100%) and VNTR 19 (2/3, 66.67%). Only VNTR 19 was not amplified from one MU-positive specimen (tissue fragment) collected from the dog lesion. Agarose gel pictures showing each amplified loci are given as supportive information **(**S2 Fig). Although MIRU1 gave two repeats (4 copies at 539 bp or 3 copies at 486 bp), only one repeat of Locus 6 (1 copy at 500 bp), VNTR 19 (2 copies at 340 bp) and ST1 (2 copies at 423 bp) was detected from the amplification of MU-positive specimens. Allelic diversity was found in MIRU1 and Locus19 resulting in the naming of two genotypes designated Z (4, 1, 2, 2) and C- (3, 1, 2, 0) from the goat and dog lesions, respectively (Table 3). The C- genotype, which is close to the human C genotype is characterized by the complete deletion of the VNTR 19 locus. Additional bands were observed from the amplification of VNTR loci of animal specimens, probably characterizing the presence of other MPMs in these samples (S1 Table).

### MIRU-VNTR profiling of animal and human lesions reveals different MU isolates

We overlapped both animal and human VNTR genotypes within the study communities to observe MU isolates distribution. Animal lesions showed different MU genotypes to those circulating in Benin, Cameroon and Ghana. The MU Agy99 human isolate used in this study as positive control confirmed the known VNTR profile C (3, 1, 2, 2). This commonly distributed African MU C genotype was also found in lesions collected from patients based in BU localities of the Nyong valley in Cameroon (Central Africa) and the Oueme valley in Benin (West Africa) (Table 3). MU isolates of animal lesions have therefore undergone genetic variations of the MIRU1 and VNTR 19 loci in the BU infected localities of Agongbo and Agbahounsou in Benin.

### Confirmation of MIRU-VNTR sequence repeats in animal samples

Discrimination of VNTR loci between animal and human lesions suggests heterogeneity of MU isolates. Repeats of amplified loci in MU-positive lesions from animals and humans were further confirmed with Sanger sequencing. Multiple Sequences Alignment (MSA) of sequenced data within NCBI-BLAST confirmed the presence of DNA compatible with MU animal specimens, sharing high similarity with previous MU isolates. MSA also showed significant sequences homology among Locus 6, ST1 and VNTR 19 loci from animal and human lesions as observed with VNTR gel-based analysis. In contrast, MIRU1 locus with sequence deposited in GenBank constituted the main discriminating and polymorphic marker between animal and human lesions from this study and published data. The MIRU1 ortholog from animal MU-positive lesion showed 99% identity to MU Agy99 complete genome, 99% to MU *subsp*. *shinshuense* DNA nearly complete genome, 99% to MU *subsp*. *shinshuense* DNA complete genome, 98% to *M*. *liflandii* 128FXT complete genome, 98% to *M*. *marinum* M complete genome, 96% to *M*. *marinum* E11 main chromosome genome and 88% to *M*. *intracellular* MOTT-64 compete genome (S3 Fig).

### Evolutionary history of MU isolates found in BU infected animals

**Fig 3:**
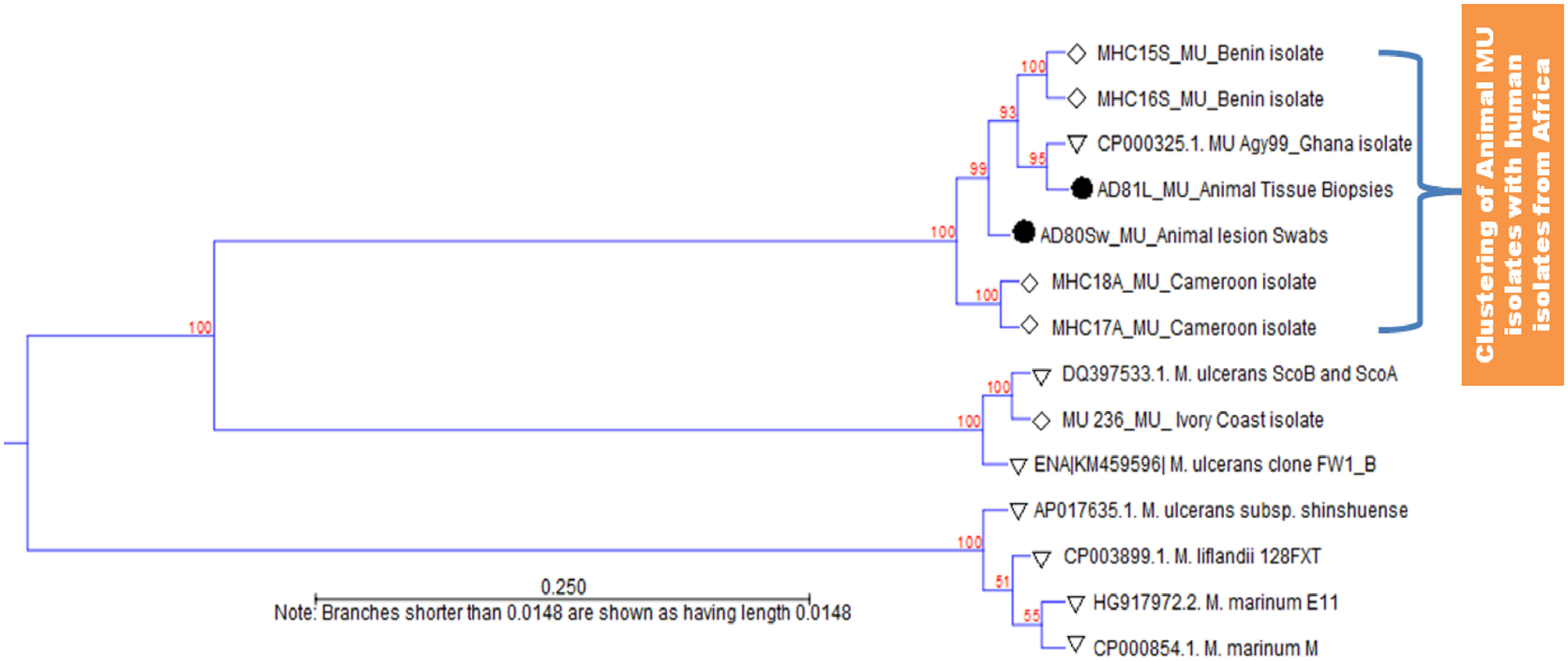
Phylogenetic reconstruction of animal and human MU isolates and comparison with reference MU strains using MIRU1 orthologs. The evolutionary history was inferred using the UPGMA method in MEGA 6. The optimal tree with the sum of branch length = 1.459786 in shown. Bootstrapping values (1000 replicates) are shown in percentage next to the branches. MIRU1 reference orthologs (white triangle) were retrieved from GenBank with accession numbers given in the tree. Sequences of animal (black circle) and human (white diamond) MU isolates are clustered at the top of the tree.

The evolutionary history of MU isolate found in animal lesions was inferred by comparison of animal and human MU isolates (data from this study) with human MU isolates from other endemic regions (Cameroon, Ivory Coast and Ghana) and published data in GenBank. The phylogenetic tree (Fig 3) revealed 2 clusters of MU isolates: one cluster of animal and human MU isolates from Africa (Cameroon, Benin, Ghana and Ivory Coast), and one cluster of MU isolates from Asia (Japan) and other Mycolatone Producing Mycobacteria (MPMs). It can be observed from this figure that human and animal MU isolates are clustered at the top of the phylogenetic tree in contrast to *M*. *marinum*, the common ancestor to mycobacterial species. Genetic comparison revealed that MU isolates from animals are less diverse and are more closely related to the strains circulating in humans in Cameroon, Benin and Ghana (Agy99). This animal strain is in contrast less closely related to the strains circulating in Ivory Coast and some parts of Ghana (ScoA and ScoB strains) (Fig 3).

## Discussion

The precise mode(s) of transmission of MU from colonized environments to humans remain unresolved and continue to be the subject of intense research. Understanding of MU ecology and transmission would greatly benefit disease control. Using the “EcoHealth” concept, which is based on a holistic approach involving humans, animals and the environment for a better understanding of BU disease, we screened domestic animals for MU-lesions found in the endemic localities of Sedje-Denou (Benin) and Akonolinga (Cameroon) and sought to source-tracked these isolates using genomics. Different groups of domestic animals were identified within the communities including cattle, carnivorous and birds. These animal groups are widespread and represent the major domesticated animals of the Old World with increased susceptibility to parasites and microbes [37,38]. Active external lesions were found on the body parts of 6.93% and 11.08% of domestic animals investigated in Benin and Cameroon respectively. Lesions mainly appeared on the head, abdomen and foot, which constitute the most exposed and susceptible parts of animals while roaming in a suspected environment. This is consistent with previous reports of BU in animals in Africa and Australia [8,11–13,17,18]. However, in BU infected patients, lesions usually appear on the legs (upper and lower) and rarely on the abdomen and head [39] as found in animals. In fact, as reported in endemic areas, protection of these parts of the body (foot, abdomen and head) reduce the susceptibility to infection by MU in colonized environments and protect against BU disease [24,28,40,41]. However, this is not the case in roaming animals. None of the lesions observed in endemic sites in Cameroon was found positive for MU DNA. However, we noticed a high number of sample positive for IS*2404* (25%), which is a preliminary confirmation of Mycolactone Producing Mycobacteria (MPMs). This suggests that instead of MU, other MPMs may colonize animal lesions as previously reported by Durnez et al. [15,42] in Benin and Dassi et al. [17] in Ivory Coast. The presence of other MPMs was further confirmed with the very low proportion of IS*2606* element and the presence of additional bands in genotyped samples. However, animal colonization by pathogens could stand as a natural event that happens while roaming and during activities like bathing, resting, drinking and food searching [18,43].

Overall, 2 active external lesions were found positive for MU in two endemic localities in Benin. The first positive lesion was observed in the abdomen of a female goat in the endemic village of Agbahounsou, while the second one was observed in the nape area of the neck of a male dog in the endemic village of Agongbo. The 2 animal lesions corresponded to type 1 lesions (< 5 cm). These BU like lesions (reddish) shared the same characteristics with those observed in small mammals in Ivory Coast and Ghana [17,18], as well as in possums, alpacas, koalas, and domestic mammals (dog, horse and cat) in Australia [8,11–13]. This is the first report of MU colonized lesions in domestic animals in Africa and outside Australia. The bacterial loads found in infected lesions were low. At the first glance, this might suggest a low quantity of the mycobacterium or a different spectrum between animal and human cases of BU. On the other hand, it could be attributed to the low quality of collected specimens (tissue fragment and wound swabs) from the animal lesions [1]. Histological sections of lesions, which were not performed in this study could have provided more details about these concerns. In fact, we realized a very fast recovery of MU infected lesions (from the female dog) just three weeks after specimens were collected. The second animal (female goat), which was unhealthy during our visit was killed by the owners due to suspicions made around its lesion as a BU infected lesion. The fast recovery of the dog’s lesion could be attributed to the treatments provided by the veterinarian doctor. It could also be due to the immunity developed by the animal organism to fight against the MU microbial pathogen. Furthermore, this might suggest a difference in the spectrum of BU disease between animals and humans. BU positive lesions found in animals were classified as type 1 which might be closely related to BU lesions of humans in Benin that are generally classified type 3 [44]. Also, the type 3 lesions are associated with mycolactone A/B, the most severe in the world [3]. In addition, the fast recovery of BU lesions found in animals could further be explained by a low toxicity of the secreted mycolactone toxin. Analysis of mycolactone profile coupled with a tentative culture of MU animal isolates could provide more information about the spectrum of BU disease between animals and humans. Culture of MU strains could further confirm the infective nature of the lesions.

We further ascertain the presence of MU through characterization of MU isolates by MIRU-VNTR genotyping. Genotype analyze of four human MU VNTR loci in animal specimens confirmed repeats corresponding to MU as previously reported in both environmental and human samples [31,33,34,45,46]. One MU genotype named Z (4, 1, 2, 2) was successfully typed in the goat BU like lesion. This Z genotype is close to the human MU genotypes (4, 1) circulating in East African Nile River Basin including Sudan, oriental DRC, and Uganda [33]. Unfortunately, Stragier and colleagues did not type the VNTR 19 and ST1 necessary to fully correlate those strains with the one found in animal lesions. Detection of East African MU strains in West Africa might suggest a strain adaptation with genetic changes in the animal, or, potential contamination from travelers from East Africa. However, investigations on the animal history did not show any relationship with individuals from this region, supporting the first hypothesis of strain adaptation which mainly affected the MIRU 1 locus. Although the Z genotype was detected in animal lesion, the C (3, 1, 2, 2) genotype circulating in West and Central Africa was confirmed in human samples from Cameroon, Benin and Ghana (reference isolate). These data are consistent with previous studies [33,47,48]. Stinear et al. [49] reported that diversity in typing markers is after high variability of tandem repeats. This might happen by insertion/deletion of nucleotides in the genome and the genome rearrangement to adapt in the new environment [32,49]. Although significant sequence homology was observed between MIRU 1 orthologs from human and animal samples, whole genome sequencing could provide more details on the genetic divergence between the two isolates. The distribution of locus 6, ST1 and VNTR 19 was constant for both human and animal specimens. MU positive lesion from the dog was negative to VNTR 19. According to Hilty et al. [32], negativity in VNTR typing can be explained by the deletion in nucleotides repetitive units which involves negative findings in PCR. Although MIRU 1 was the main determinant of MU genotypes between animals and humans, locus 6 and VNTR 19 have been suggested to discriminate between MU strains from humans [31,34,47]. However, these considerations could vary when comparing MU isolates of human and environmental samples [18,32,46]. In a recent study, Narh et al. [18] described four similar MU genotypes (W, X, Y and Z) between human and infected water bodies. This further confirms that slow water bodies which serve as vehicles for the dissemination of MU strains [50] also stand as important sources of MU infection in humans [18]. The environmental contamination of animals by MU colonized water bodies might also happen if considering the same microhabitat and anatomy they share with humans. In addition, animals like dogs usually bath in these water bodies as humans. This might explain why the C- (3, 1, 2, 0) genotype found in the infected dog is close to the human genotype C circulating in the same region. This strain of MU might have undergone complete deletion/modification of VNTR 19 as some human strains (FS3 and FS6) described in Ghana by [18]. The C- genotype detected in the animal lesion has previously been detected in a human MU positive lesion (2084) in Ivory Coast and was assigned a genotype C by the authors [48]. This further ascertains the close similarity between human and animal isolates of MU. Solange et al. [48] also showed that human MU isolates can harbor partial repeats, while Narh et al. [18] reported the distribution of new repeats in other MPMs. Heterogeneity among VNTR loci highlights the necessity to combine all four loci to match MU isolates of human and environmental samples in transmission studies. The low susceptibility of small mammals and domestic animals to MU infection highlights the mystery behind the transmission of BU in endemic areas. Although inter-human transmission of BU remains a rare event, the colonization of domestic mammals by MU emphasizes the risks associated with the close contact between humans and these animals. Additional attention should be paid in manipulating animals in endemic areas. Furthermore, MU infection should be suspected in animals domiciled in endemic areas and exhibiting ulcerative skin lesions [12].

Sequencing of amplified loci further confirms the positivity of animal lesions with high sequence homology to MU orthologs in GenBank. This data is consistent with previous studies of human and environmental MU samples [18,45,49]. Overall sequenced data were more related to MU strains than other MPMs. Only specific bands from agarose gel electrophoresis were cut and sequenced, leaving behind the non-specific bands probably corresponding to other MPMs as reported by Narh et al. [18]. The evolutionary history of animal MU isolates was inferred in comparison with human MU isolates from the same locality and reference MU orthologs from NCBI-GenBank. Phylogenetic analysis clustered animal and human samples, releasing the other MPMs at the bottom of the tree (probably the less evaluated). These data further highlight the similarity between animal and human MU strains and might suggest a common contamination source for both organisms. Clustering of environmental MU isolates with human isolates has significantly been reported and is the main thought of linking human BU cases with MU colonized environments [18,45,49]. Understanding the trophic relationship between MU colonized matrices is seminal to understanding its transmission mode(s) from the infected environment to humans and animals. Several environmental matrices are susceptible to MU and two main hypotheses have been postulated on the transmission mode of MU including indirect bites from insects (aquatic insects and mosquitoes) and direct inoculation of the bacilli into the skin through trauma [10,51–53]. For any of these postulates, possibilities of animal and human colonization by MU could be set at the same level, as they share the same microhabitat, they all have contact with the risk environments (infected water bodies, biofilms, soils and faeces) and they are all susceptible to insect bites (aquatic insects and mosquitoes). In some circumstances, small mammals like possums, shrews, rats, etc., could be more exposed because of their unlimited displacements in the colonized environment. However, the number of BU cases reported in this group of animals remains very low in endemic areas comparing to human reported cases (8,11,12, 18,19,42]. This might suggest that human beings represent the main host with high spectrum for MU replication. The ‘EcoHealth’ concept which considers both the ecology and health of all living organisms has been pointed out in this study to better understand how human and animal get infected from the colonized environment. Even if infected animals and humans share different MU isolates; we believe that they share a common source of contamination and that the strains have undergone a host adaptation in animal as genetically reported by Stinear et al. [49]. It is postulated that small mammals could directly infect humans with MU through animal bites, or indirectly through food and water contamination or handling during hunting [10]. The susceptibility of domestic animals to MU as described in this study is a great public health concern that further highlights the risks associated with the manipulation of animals in BU endemic areas. Control measures should therefore be undertaken to alert the risk populations and additional studies should be implemented to understand the definite role of animal hosts in BU transmission. This study suggests the paradigm of MU as an environmental pathogen involved in different transmission pathways depending on its location. Understanding MU ecology in its respective, different endemic loci will support our knowledge about the diseases transmission and help with infection control.

### Conclusion

This study describes the first report of MU colonized lesions in domestic animals in Africa. Two animal lesions from one goat and one dog were found to be infected with MU in the villages of Agbahounsou and Agongbo which are major endemic foci for human BU in Southern Benin. VNTR typing of infected lesions revealed two different genotypes, a Z genotype from the goat lesion and a C- genotype from the dog lesion. We overlapped both animal and human VNTR genotypes within the study communities to observe MU isolates distribution. The data revealed a divergence between the animal genotypes and the human C genotype circulating in the investigated areas. Source-tracking of MU sequences between animals and humans revealed close homology among loci orthologs from this study and previous studies. In addition, phylogenetic analysis clustered animal and human samples. This might suggest a common source of contamination which could be followed by a strain rearrangement to adapt into the new environment. Domestic hosts which are in close contact with populations could play a role in the transmission cycle of MU as previously reported with small mammals. For control measures, MU infection should be suspected in domestic hosts living near humans in BU endemic areas and presenting with ulcerative skin disease.

## Supporting information

S1 Fig Suspected BU like lesions that were found negative to MU molecular targets (IS*2404*, IS*2606* and KR-B) after qPCR analysis and characterization by MIRU-VNTR genotyping. (ZIP)

S2 Fig Agarose gel picture showing MIRU-VNTR typing of animal and human MU ecovars. **A**: MIRU1; **B**: Locus 6; **C**: ST1; **D**: VNTR-19; **M**: 100 bp-DNA ladders; **Cp**: DNA Positive control (MU Agy99, Ghana); **CN**: Negative contro; **MHC15S et MHC16S**: Human clinical samples from Benin (Sedje health center); **MHC17A et MHC18A**: Human clinical samples from Cameroon (Akonlinga district hospital); **AD80Sw et AD81L**: Respectively wound swab and tissue fragment from MU-infected goat lesion; **AD01L**: Tissue fragment from MU-infected dog lesion (This sample was negative to VNTR 19).
(RAR)

S3 Fig Displaying of NCBI-BLAST datasheet after alignment of MU consensus sequence from animal lesion with reference sequences in GeneBank. **A)** Flow chart of reference MU isolates sharing sequence homology with animal MU isolate. **B)** Percentage identity between animal MU sequence and reference sequences in GenBank.
(RAR)

S1 Table Distribution of MIRU-VNTR loci in MU-positive and negative animal lesions. Bp: Base pair; UA: Unassigned; Allelic genotypes were attributed according to the copy numbers of each amplified locus (**Stragier et al., 2005**). Unknown DNA bands length were unassigned a specific copy number and a specific genotype.
(PPTX)
