# Supplementary figures and images for: Domestic animals infected with Mycobacterium ulcerans—Implications for transmission to humans
Source: PLoS Negl Trop Dis. 2018 Jul 2;12(7):e0006572. doi: 10.1371/journal.pntd.0006572 (PMC6044547; doi:10.1371/journal.pntd.0006572)

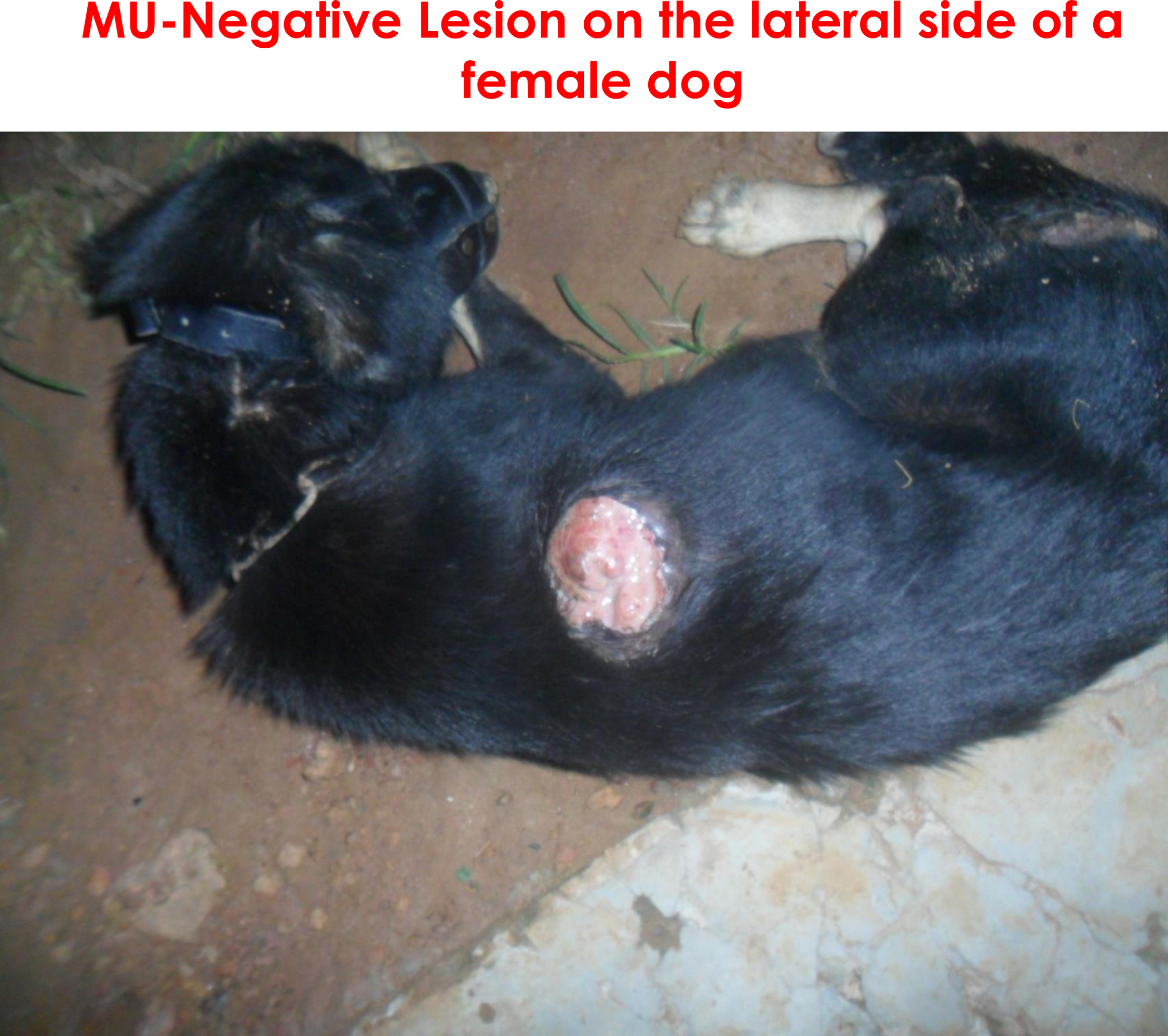

Supplement: S1 Fig — (ZIP) [file pntd.0006572.s001.zip › PACE Corrected/S1. Fig. MU-Negative lesions in DAs-1.tif]

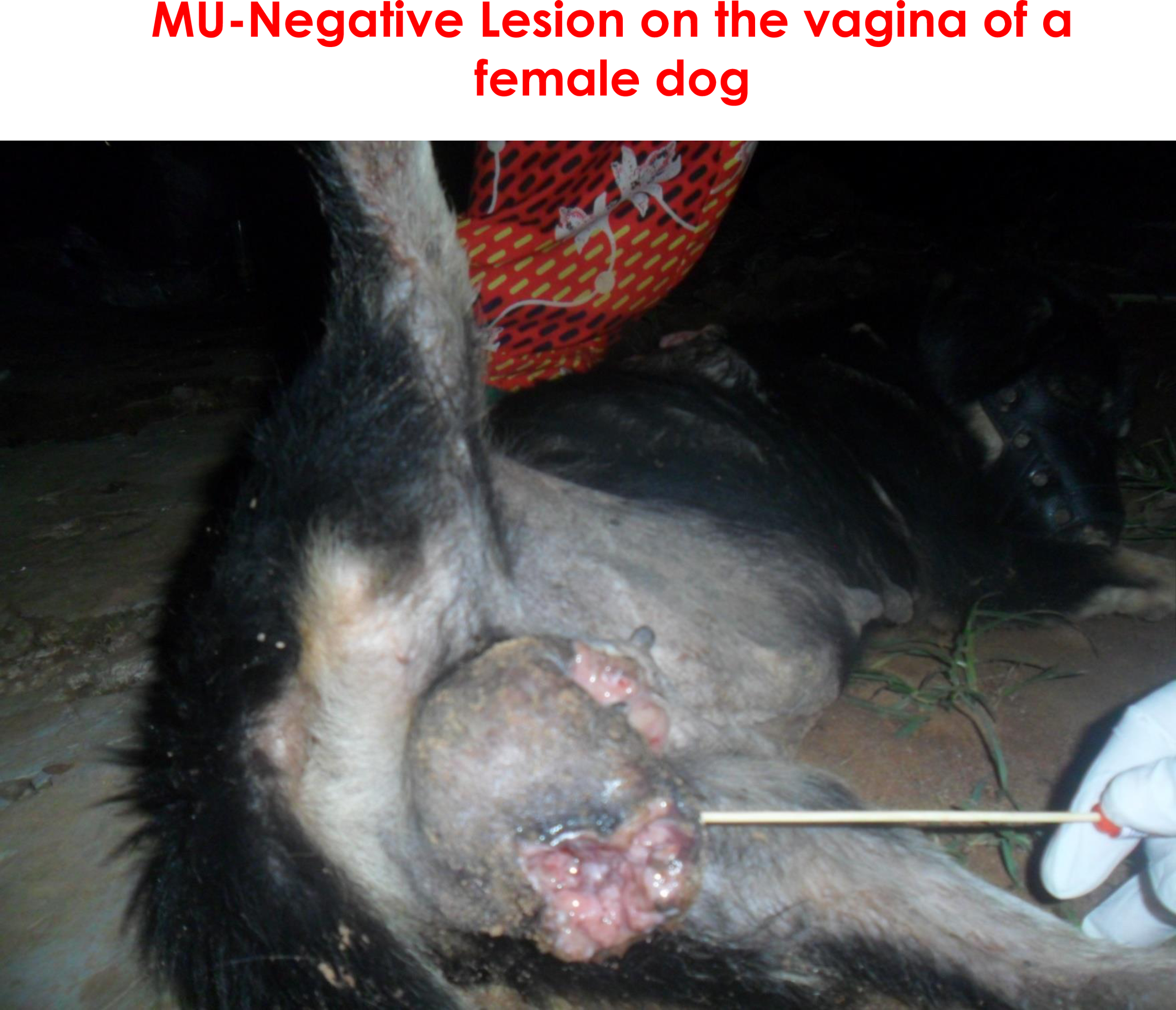

Supplement: S1 Fig — (ZIP) [file pntd.0006572.s001.zip › PACE Corrected/S1. Fig. MU-Negative lesions in DAs-2.tif]

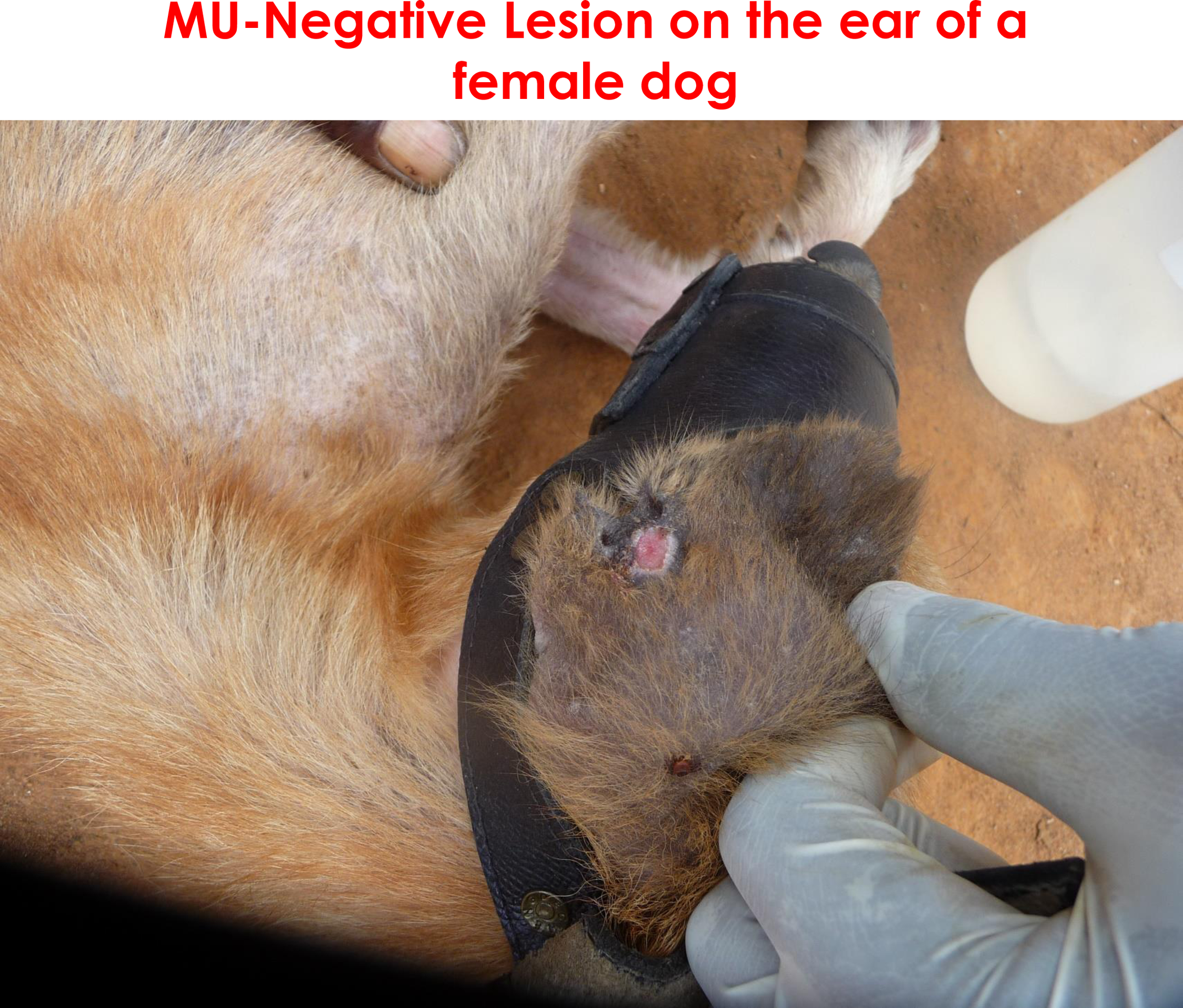

Supplement: S1 Fig — (ZIP) [file pntd.0006572.s001.zip › PACE Corrected/S1. Fig. MU-Negative lesions in DAs-3.tif]

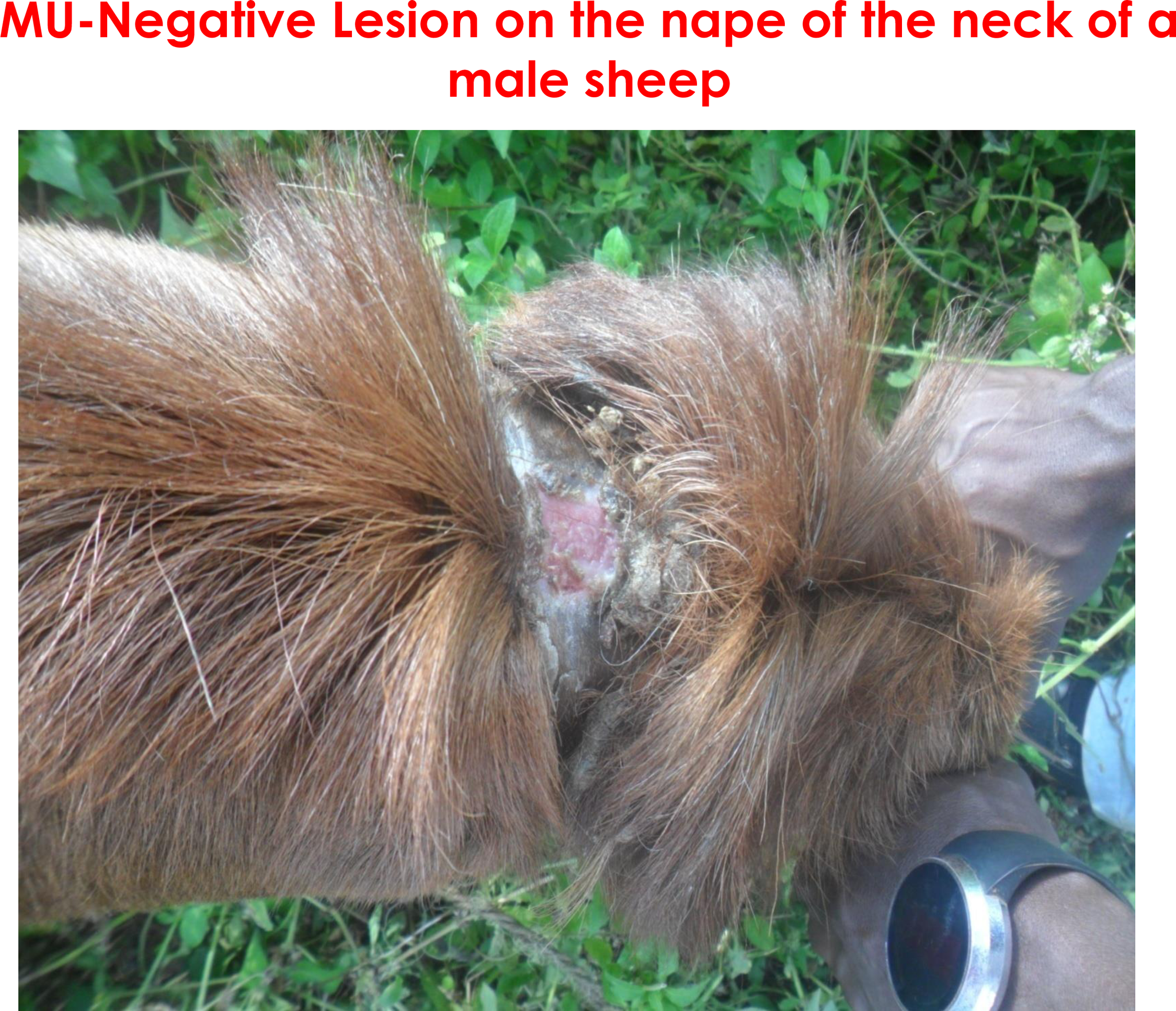

Supplement: S1 Fig — (ZIP) [file pntd.0006572.s001.zip › PACE Corrected/S1. Fig. MU-Negative lesions in DAs-4.tif]

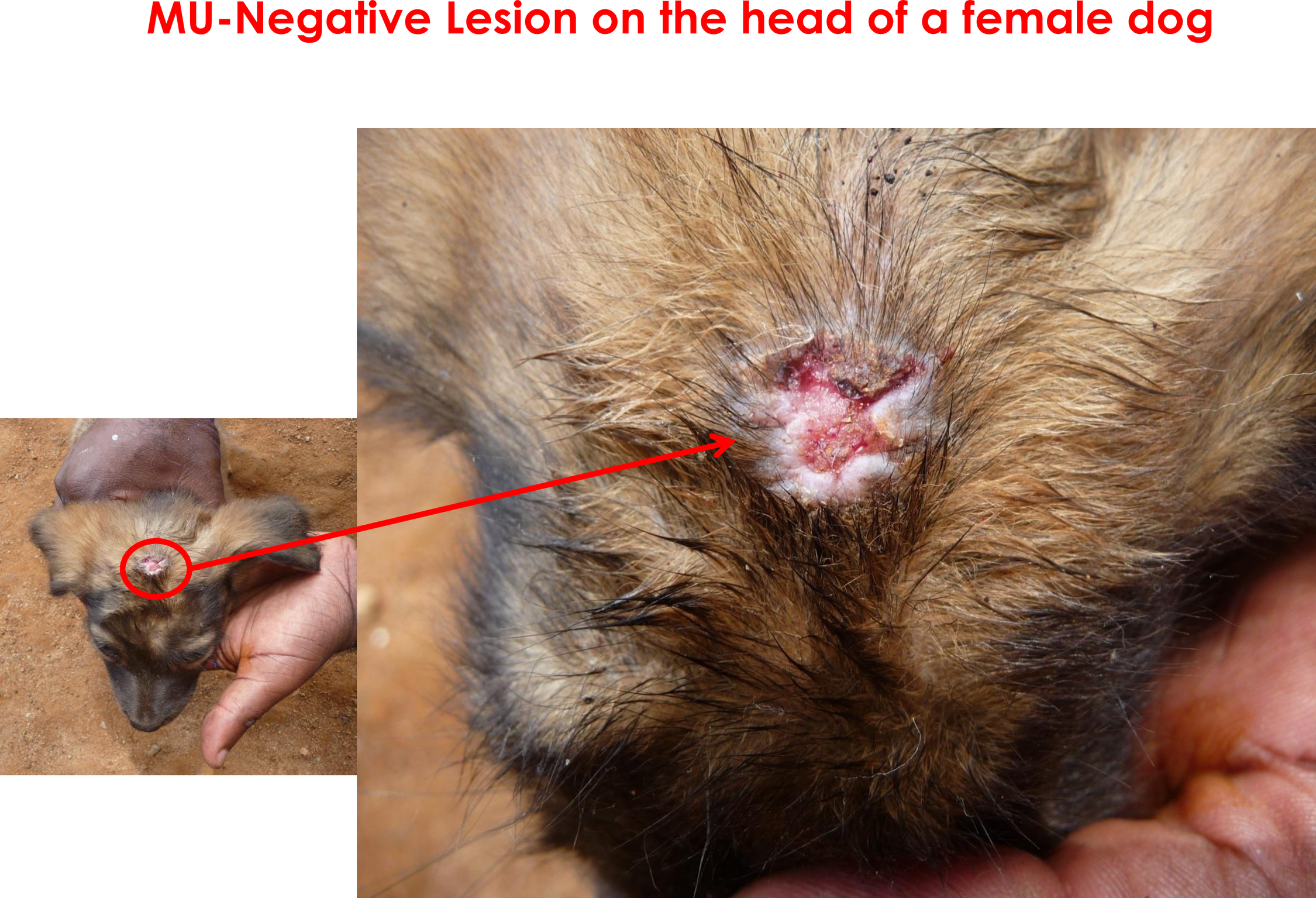

Supplement: S1 Fig — (ZIP) [file pntd.0006572.s001.zip › PACE Corrected/S1. Fig. MU-Negative lesions in DAs-5.tif]

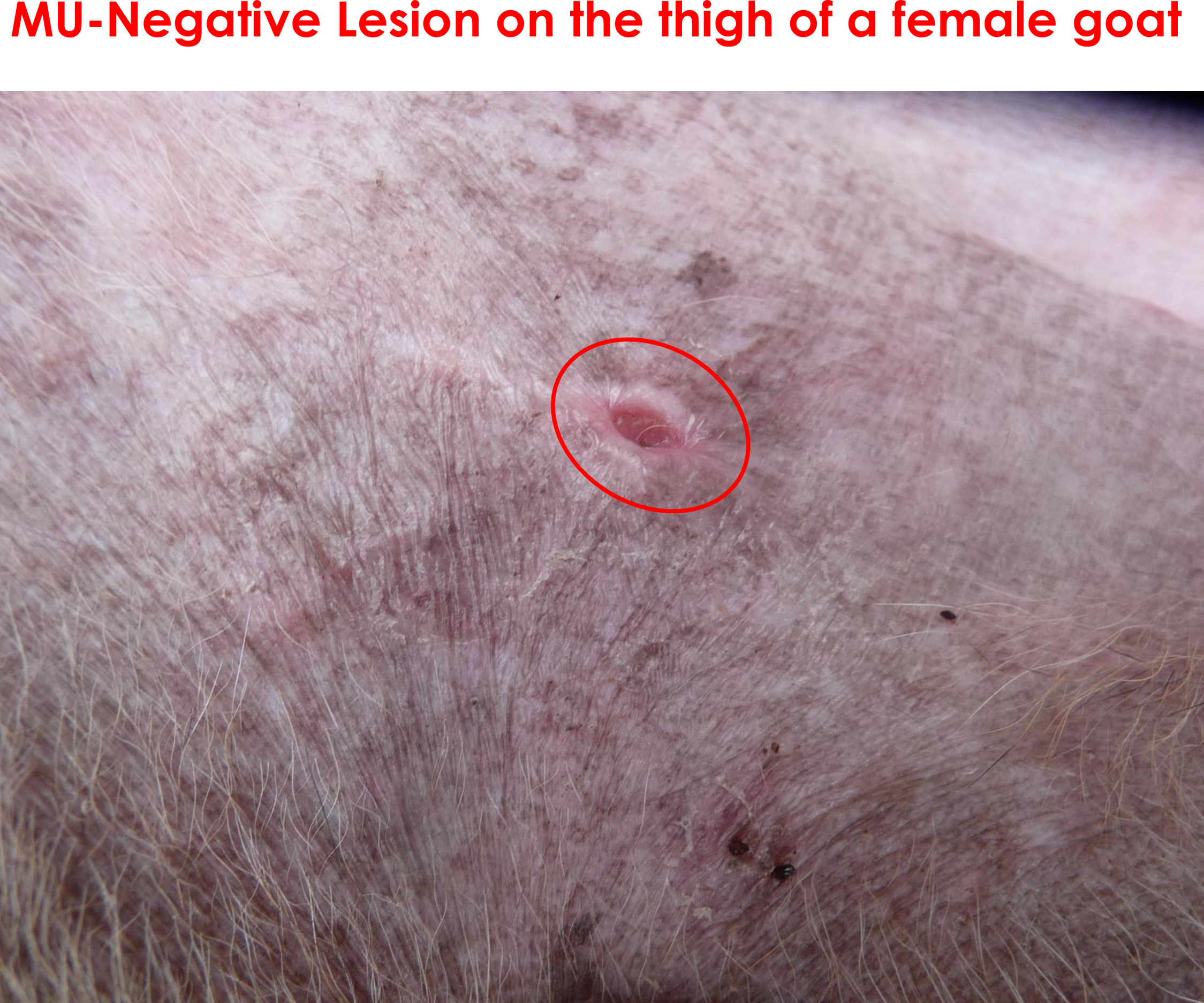

Supplement: S1 Fig — (ZIP) [file pntd.0006572.s001.zip › PACE Corrected/S1. Fig. MU-Negative lesions in DAs-6.tif]

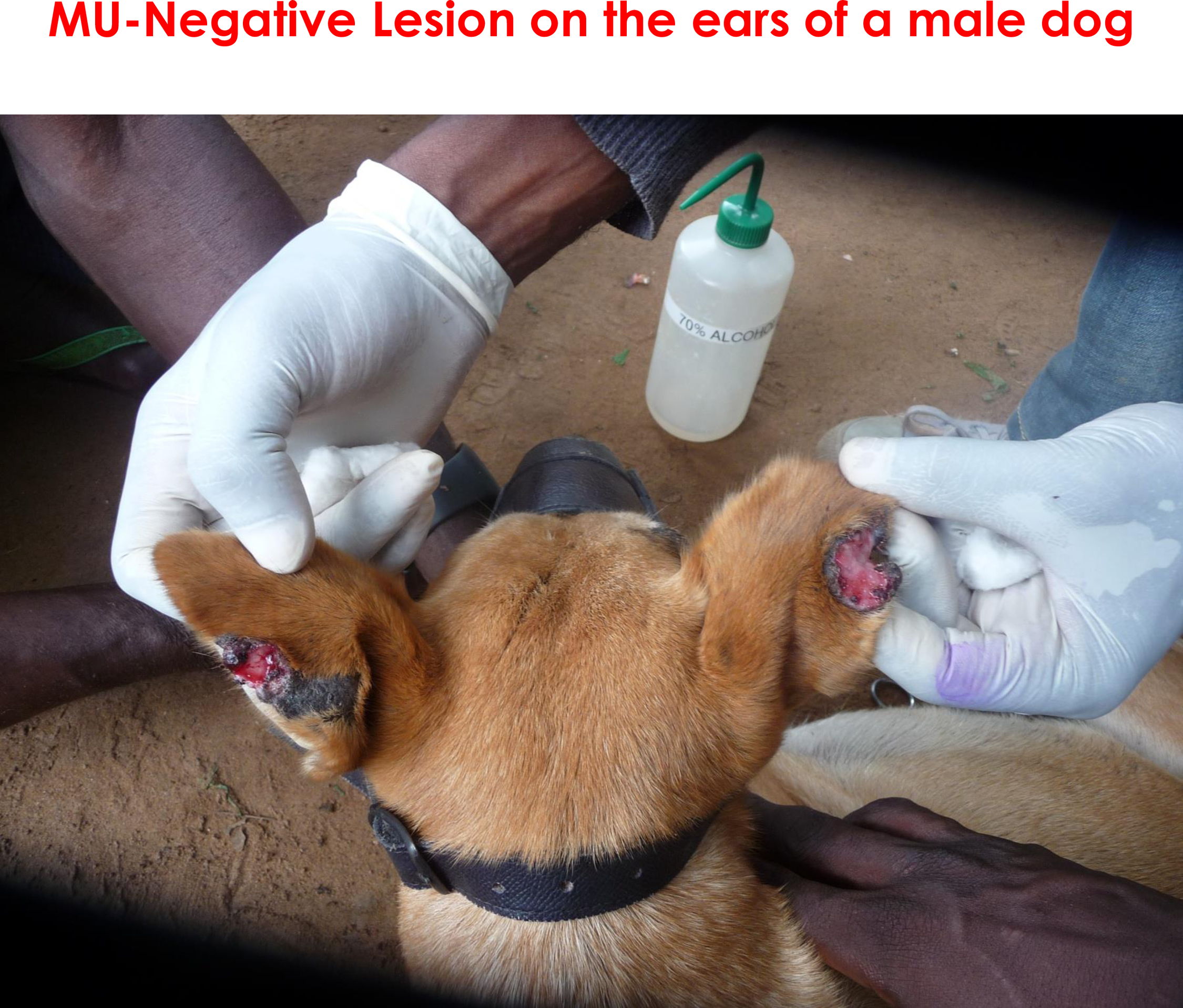

Supplement: S1 Fig — (ZIP) [file pntd.0006572.s001.zip › PACE Corrected/S1. Fig. MU-Negative lesions in DAs-7.tif]
