# Supplementary material for: Domestic animals infected with Mycobacterium ulcerans—Implications for transmission to humans
Source: PLoS Negl Trop Dis. 2018 Jul 2;12(7):e0006572. doi: 10.1371/journal.pntd.0006572 (PMC6044547; doi:10.1371/journal.pntd.0006572)
Supplement: S1 Table — Bp: Base pair; UA: Unassigned; Allelic genotypes were attributed according to the copy numbers of each amplified locus (Stragier et al., 2005). Unknown DNA bands length were unassigned a specific copy number and a specific genotype. (PPTX) [file pntd.0006572.s004.pptx]

## Slide 1
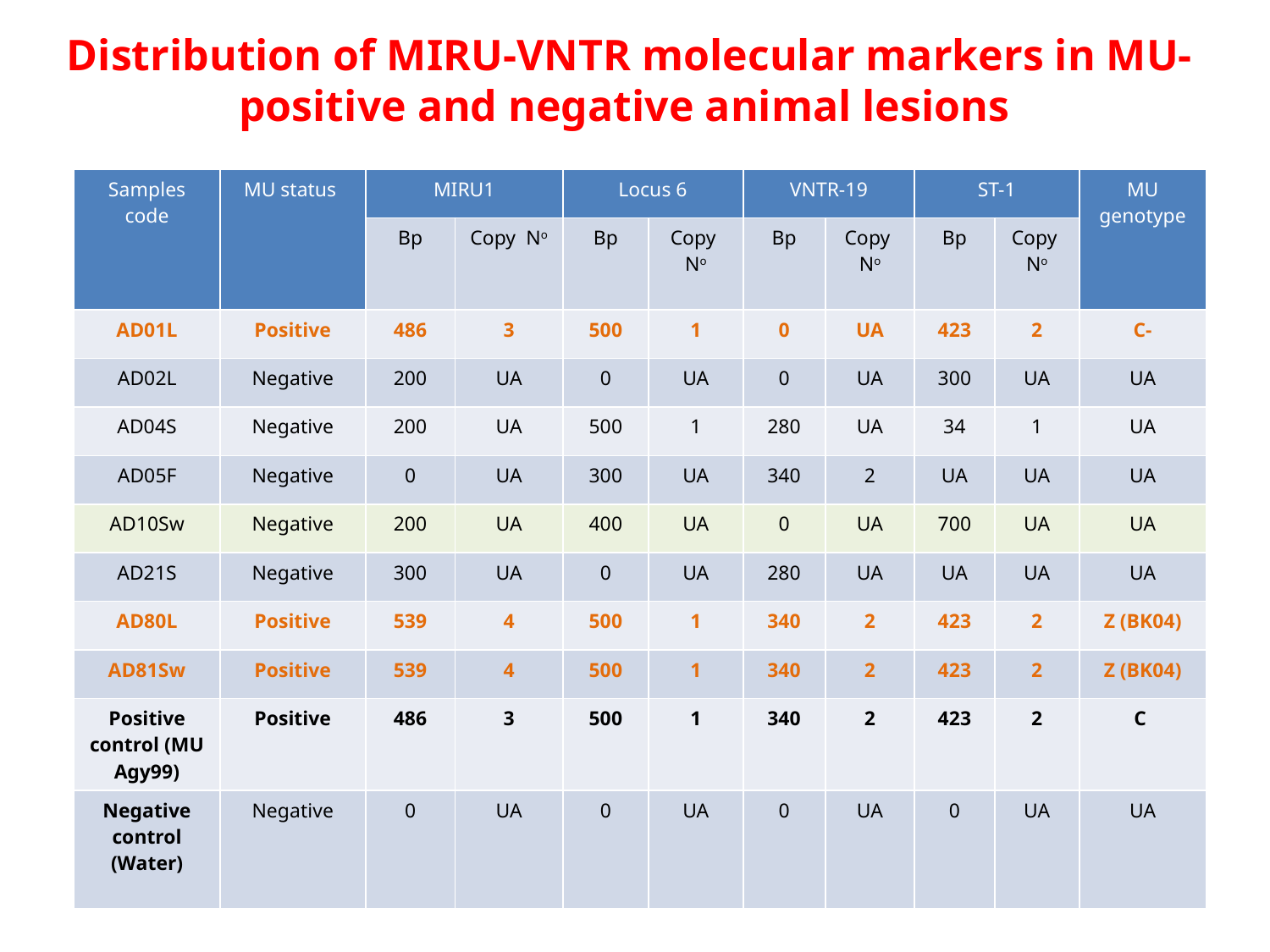

Distribution of MIRU-VNTR molecular markers in MU-positive and negative animal lesions
| Samples code | MU status | MIRU1 | | Locus 6 | | VNTR-19 | | ST-1 | | MU genotype |
| --- | --- | --- | --- | --- | --- | --- | --- | --- | --- | --- |
| | | Bp | Copy No | Bp | Copy No | Bp | Copy No | Bp | Copy No | |
| AD01L | Positive | 486 | 3 | 500 | 1 | 0 | UA | 423 | 2 | C- |
| AD02L | Negative | 200 | UA | 0 | UA | 0 | UA | 300 | UA | UA |
| AD04S | Negative | 200 | UA | 500 | 1 | 280 | UA | 34 | 1 | UA |
| AD05F | Negative | 0 | UA | 300 | UA | 340 | 2 | UA | UA | UA |
| AD10Sw | Negative | 200 | UA | 400 | UA | 0 | UA | 700 | UA | UA |
| AD21S | Negative | 300 | UA | 0 | UA | 280 | UA | UA | UA | UA |
| AD80L | Positive | 539 | 4 | 500 | 1 | 340 | 2 | 423 | 2 | Z (BK04) |
| AD81Sw | Positive | 539 | 4 | 500 | 1 | 340 | 2 | 423 | 2 | Z (BK04) |
| Positive control (MU Agy99) | Positive | 486 | 3 | 500 | 1 | 340 | 2 | 423 | 2 | C |
| Negative control (Water) | Negative | 0 | UA | 0 | UA | 0 | UA | 0 | UA | UA |
